# Selected SNARE proteins are essential for the polarized membrane insertion of igf-1 receptor and the regulation of initial axonal outgrowth in neurons

**DOI:** 10.1038/celldisc.2015.23

**Published:** 2015-09-01

**Authors:** Diego Grassi, Florentyna Bustos Plonka, Mariana Oksdath, Alvaro Nieto Guil, Lucas J Sosa, Santiago Quiroga

**Affiliations:** 1 Departamento de Química Biológica-CIQUIBIC, Facultad de Ciencias Químicas, Universidad Nacional de Córdoba-CONICET, Córdoba, Argentina

**Keywords:** Neuronal differentiation, neuronal polarization, membrane expansion, IGF-1 receptor, VAMP4, SNAP23, Sintaxyn6.

## Abstract

The establishment of polarity necessitates initial axonal outgrowth and, therefore, the addition of new membrane to the axon’s plasmalemma. Axolemmal expansion occurs by exocytosis of plasmalemmal precursor vesicles (PPVs) primarily at the neuronal growth cone. Little is known about the SNAREs family proteins involved in the regulation of PPV fusion with the neuronal plasmalemma at early stages of differentiation. We show here that five SNARE proteins (VAMP2, VAMP4, VAMP7, Syntaxin6 and SNAP23) were expressed by hippocampal pyramidal neurons before polarization. Expression silencing of three of these proteins (VAMP4, Syntaxin6 and SNAP23) repressed axonal outgrowth and the establishment of neuronal polarity, by inhibiting IGF-1 receptor exocytotic polarized insertion, necessary for neuronal polarization. In addition, stimulation with IGF-1 triggered the association of VAMP4, Syntaxin6 and SNAP23 to vesicular structures carrying the IGF-1 receptor and overexpression of a negative dominant form of Syntaxin6 significantly inhibited exocytosis of IGF-1 receptor containing vesicles at the neuronal growth cone. Taken together, our results indicated that VAMP4, Syntaxin6 and SNAP23 functions are essential for regulation of PPV exocytosis and the polarized insertion of IGF-1 receptor and, therefore, required for initial axonal elongation and the establishment of neuronal polarity.

## Introduction

The formation of a polarized neuron, containing one long axon and several branching dendrites, requires the action of two interrelated processes, specification of the axon and neurite outgrowth. The initial signals and pathways that determine polarity by regulating initial axonal outgrowth are at the present starting to be understood. A particularly early event, in neurons that do not yet exhibit a discernible axon (stage 2 of differentiation, [1]) is the segregation of activatable, membrane inserted, IGF-1 receptors to one neurite. Subsequently, phosphatidylinositol-3 kinase is activated and its product, PIP_3,_ accumulate in the distal region and growth cone of that neurite, together with the IGF-1 receptor. These events are critical for the outgrowth of the future axon [1–4]. Besides axonal specification, the establishment of polarity necessitates initial axonal outgrowth and, therefore, the addition of new membrane to the axon’s plasmalemma. Axolemmal expansion occurs by exocytosis of plasmalemmal precursor vesicles (PPVs) primarily at the neuronal growth cone [5–7], a process regulated by IGF-1 activation of the phosphatidylinositol-3 kinase pathway [8–10]. Exocytosis requires two different processes, vesicle docking or attachment and vesicle fusion [11, 12]. The underlying mechanisms are mediated by different families of proteins. Docking and targeting is often regulated by the exocyst complex involved in neurite outgrowth and synaptogenesis in neurons, but not in synaptic vesicle fusion [11, 13]. Previous published data from our laboratory indicated that, in cultured hippocampal neurons, TC10 activation by IGF-1 and recruitment of exo70 to the growth cone plasmalemma are necessary for the regulation of PPVs exocytosis and, therefore, initial axonal outgrowth and the establishment of neuronal polarity [14]. Vesicle fusion, in turn, is primarily a function of the SNARE family proteins [11, 12]. Although the role and identity of the SNARE proteins in the fusion of synaptic vesicles have been extensively studied [15, 16], little is known about the SNAREs involved in the regulation of PPV fusion with the neuronal plasmalemma, even if it has been shown that some of them, such as SNAP25, Syntaxin1 and VAMP7 seem to be required for axonal growth *in vitro* [17–19]. In this context the experiments shown here were designed to answer the following questions: (i) is there a specific set of SNARE proteins involved in the regulation of PPV exocytosis at early stages of neuronal differentiation and necessary for initial axonal growth and the establishment of neuronal polarity? And (ii) is this select group of SNARE proteins also necessary for the polarized exocytosis of IGF-1 receptor-containing vesicles in the growth cones of the future axon? We selected seven SNARES which seem to be involved in neurite outgrowth: VAMP4 [20, 21], VAMP7 [19], Syntaxin1 [22], Syntaxin6 [23], SNAP23 [21, 24], SNAP25 [17, 18] and VAMP2 (primarily involved in axonal guidance [25] and synaptic function [26, 27] but apparently not in neural elongation [18] in hippocampal pyramidal neurons. However, it has been shown that VAMP2 can be involved in neurite elongation in cortical neurons containing Apo4-Mito or FP4-Mito growing on laminin [28]. Our results show that five out of these seven SNARE proteins (VAMP2, VAMP 4, VAMP7, Syntaxin6 and SNAP23) are expressed by hippocampal pyramidal neurons before polarization. Expression silencing of three of these proteins (VAMP4, Syntaxin6 and SNAP23) repressed axonal outgrowth and the establishment of neuronal polarity, by inhibiting IGF-1 receptor exocytotic polarized insertion, necessary for neuronal polarization [1]. Moreover, stimulation with IGF-1 triggered the association of VAMP4, Syntaxin6 and SNAP23 to vesicular structures carrying the IGF-1 receptor and overexpression of a negative dominant form of Syntaxin6 significantly inhibited exocytosis of IGF-1 receptor containing vesicles at the neuronal growth cone. Taken together, our results indicate that VAMP4, Syntaxin 6 and SNAP23 function are essential for regulation of PPV exocytosis and the polarized insertion of IGF-1 receptor and, therefore, required for initial axonal elongation and the establishment of neuronal polarity.

## Results

**Figure 1:**
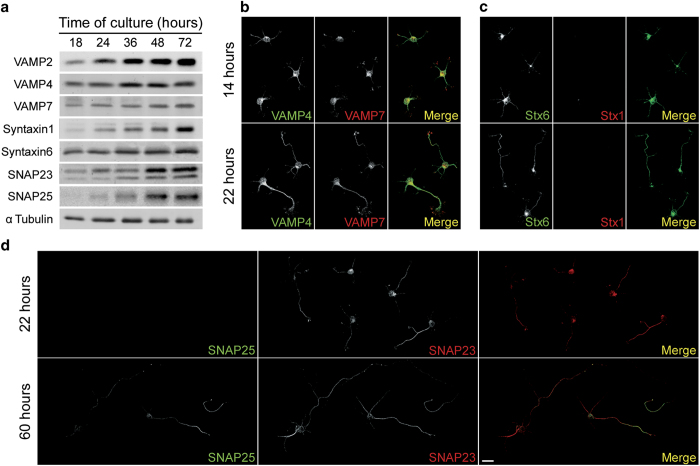
(**a**) Western blot showing the expression of VAMP2 (first row; apparent molecular weight 18 kDa), VAMP4 (second row; apparent molecular weight 16 kDa), VAMP7 (third row; apparent molecular weight 24 kDa), Syntaxin1 (fourth row; apparent molecular weight 34 kDa), Syntaxin6 (fifth row; apparent molecular weight 31 kDa), SNAP23 (sixth row; apparent molecular weight 23 kDa) and SNAP25 (seventh row; apparent molecular weight 25 kDa),) in hippocampal pyramidal neurons after 18, 24, 36, 48 or 72 h of DIV. Tubulin was used as a loading control. A particularity of SNAP23 was that, besides the expected band (apparent molecular weight 23 kDa) a less intense, lower band was observed. This is probably not due to nonspecific labeling since this band was observed in neurons in culture, but not always in brain preparations. This double-band pattern has been previously described and may represent cleavage products, alternative splicing, post-translational modifications [55] or SNAP23 isoforms [56]. (**b**) Double immunofluorescence micrographs showing the distribution of VAMP4 (green) and VAMP7 (red) in hippocampal pyramidal neurons in culture after 14 (top) or 22 (bottom) h of DIV. (**c**) Double immunofluorescence micrographs showing the distribution of Syntaxin6 (green) and Syntaxin1 (red) in hippocampal pyramidal neurons in culture after 14 (top) or 22 (bottom) h of DIV. (**d**) Double immunofluorescence micrographs showing the distribution of SNAP25 (green) and SNAP23 (red) in hippocampal pyramidal neurons in culture after 22 (top) or 60 (bottom) h of DIV. Calibration bar= 20 μm.

A prerequisite for a protein to be involved in neuronal polarization would be to be expressed early before this phenomenon occurs (in our system most cells exhibit a discernible axon at 20–24 h in culture, so we selected SNARE proteins expressed after 18 h in culture). Results showed that five of the preselected proteins (VAMP2, VAMP4, VAMP7, Sintaxyn6 and SNAP23) are expressed after 18 h in culture. In contrast, both Syntaxin1 and SNAP25 are expressed above detection levels only after 24–36 h in culture (Figure 1a). We also analyzed the expression and distribution of VAMP4, VAMP7, Syntaxin1, Sintaxin6, SNAP23 and SNAP25 in primary cultures of hippocampal neurons at 14 or 22 h of differentiation *in vitro*. Consistent with the data shown in Figure 1a the expression of VAMP4 and VAMP7 is similar in neurons after both 14 and 22 h of DIV (Figure 1b). Immunostaining with Sintaxin1 and Syntaxin6 showed that the expression of the former is below detection levels after 14 h of DIV and only evident after 22 h of DIV in already polarized neurons. In contrast, there is a relatively prominent expression of Syntaxin6 after 14 h of DIV (Figure 1c). Regarding SNAP23 and SNAP25, we observed a robust expression of the former after 22 h of DIV but the latter is below detection levels at that time and expressed only after 60 h of DIV in neurons exhibiting relatively long axons (Figure 1d). Supplementary Figure S1 shows that VAMP2, VAMP4, VAMP7, Syntaxin6 and SNAP23 were expressed in neurons at early stage 3 of differentiation and enriched at the axons and growth cones, prominent sites for the addition of new membrane in developing neurons [5, 8, 9, 29].

**Figure 2:**
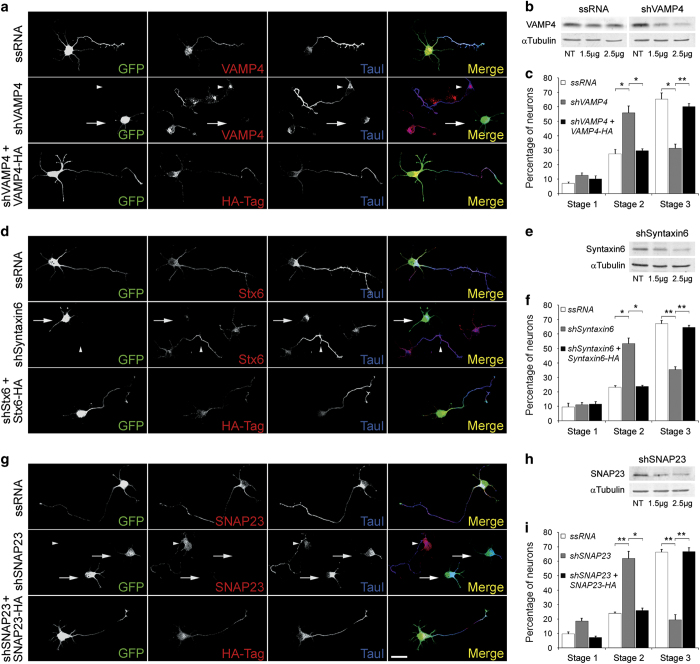
(**a**) (first two rows) Double immunofluorescence micrographs of hippocampal neurons after 24 h in culture that show the localizations of VAMP4 (red) or tau-1 (blue) and GFP as a transfection marker. Note that the neurons transfected with VAMP4-targeted shRNA (second row, arrows) did not develop axons and did not target tau-1 to any particular neurite. Non-transfected neurons in the same culture exhibited tau-1- and VAMP4-positive axons (arrowheads). (bottom) Co-transfection with VAMP4-targeted shRNA plus wild-type, HA-tagged VAMP4 (red) rescued the phenotype. Outgrowth of a tau-1- positive axon (blue) is evident. (**b**) Western blots showing VAMP4 levels in rat brain glioma cells C6 transfected with ssRNA (left) and a VAMP4-targeted shRNA (right). Tubulin was used as a loading control. (**c**) Relative percentages (±s.e.m.) of control neurons or neurons containing VAMP4-targeted shRNA at specific stages of differentiation after 36 h in culture. *n*=3 independent experiments. At least 100 neurons were scored for each condition. (**d**) (first two rows) Double immunofluorescence micrographs of hippocampal neurons after 24 h in culture that show the localizations of Syntaxin6 (red) or tau-1 (blue) and GFP as a transfection marker. Note that the neurons transfected with Syntaxin6-targeted shRNA (second row, arrows) did not develop axons and did not target tau-1 to any particular neurite. Non-transfected neurons in the same culture exhibited tau-1- and Syntaxin6-positive axons (arrowheads). (bottom) Co-transfection with Syntaxin6-targeted shRNA plus wild-type, HA-tagged Syntaxin6 (red) rescued the phenotype. Outgrowth of a tau-1- positive axon (blue) is evident. (**e**) Western blots showing Syntaxin6 levels in rat brain glioma cells C6 cultured in the presence of a Syntaxin6-targeted shRNA. Tubulin was used as a loading control. (**f**) Relative percentages (±s.e.m.) of control neurons or neurons containing Syntaxin6-targeted shRNA at specific stages of differentiation after 36 h in culture. *n*=3 independent experiments. At least 100 neurons were scored for each condition. (**g**) (first two rows) Double immunofluorescence micrographs of hippocampal neurons after 24 h in culture that show the localizations of SNAP23 (red) or tau-1 (blue) and GFP as a transfection marker. Note that the neurons transfected with SNAP23-targeted shRNA (second row, arrows) did not develop axons and did not target tau-1 to any particular neurite. Non-transfected neurons in the same culture exhibited tau-1- and SNAP23-positive axons (arrowheads). (bottom) Co-transfection with SNAP23-targeted shRNA plus wild-type, HA-tagged SNAP23 (red) rescued the phenotype. Outgrowth of a tau-1- positive axon (blue) is evident. (**h**) Western blots showing SNAP23 levels in rat brain glioma cells C6 cultured in the presence a SNAP23-targeted shRNA (right). Tubulin was used as a loading control. (**i**) Relative percentages (±s.e.m.) of control neurons or neurons containing VAMP4-targeted shRNA at specific stages of differentiation after 36 h in culture. *n*=3 independent experiments. At least 100 neurons were scored for each condition. *P⩽ 0.005, **P⩽ 0.001. Calibration bar= 20 μm.

**Figure 3:**
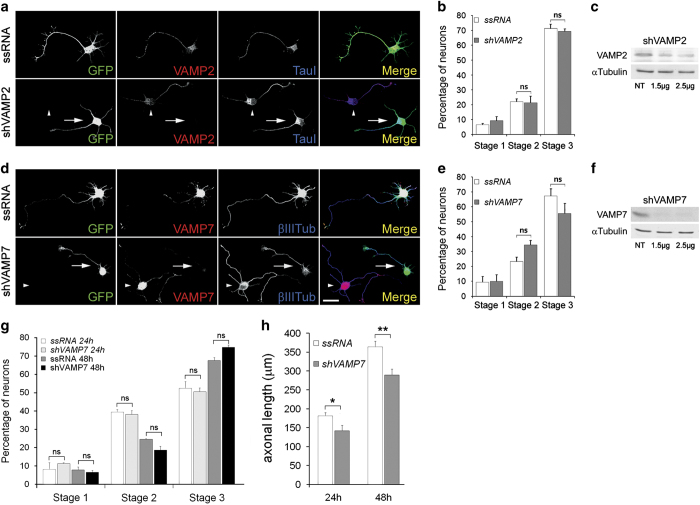
(**a**) Double immunofluorescence micrographs of hippocampal neurons after 24 h in culture that show the localizations of VAMP2 (red) or tau-1 (blue) and GFP as a transfection marker. Note that both the cells transfected with an ssRNA (top) and a VAMP2-targeted shRNA (bottom) exhibit an axon-like process enriched in tau-1. (**b**) Relative percentages (+ s.e.m.) of control neurons or neurons containing VAMP2-targeted shRNA at specific stages of differentiation after 36 h in culture. *n*=3 independent experiments. At least 100 neurons were scored for each condition. (**c**) Western blots showing VAMP2 levels in rat brain glioma cells C6 cultured in the presence a VAMP2-targeted shRNA (right). Tubulin was used as a loading control. (**d**) Double immunofluorescence micrographs of hippocampal neurons after 24 h in culture that show the localizations of VAMP7 (red) or tau-1 (blue) and GFP as a transfection marker. Note that both the cells transfected with a ssRNA (top) or a VAMP7-targeted shRNA (bottom) exhibit an axon-like process enriched in tau-1. (**e**) Relative percentages (+ s.e.m.) of control neurons or neurons containing VAMP7-targeted shRNA at specific stages of differentiation after 36 h in culture. *n*=3 independent experiments. At least 100 neurons were scored for each condition. (**f**) Western blots showing VAMP7 levels in rat brain glioma cells C6 cells cultured in the presence a VAMP7-targeted shRNA (right). Tubulin was used as a loading control. (**g**) Relative percentages (± s.e.m.) of control neurons or neurons containing VAMP7-targeted shRNA at specific stages of differentiation after 24 h or 48 h in culture on laminin-coated cover glasses. *n*=3 independent experiments. At least 50 neurons were scored for each condition. (**h**) Axonal length (± s.e.m. of control neurons or neurons containing VAMP7-targeted shRNA at specific stages of differentiation after 24 h or 48 in culture. *n*=3 independent experiments. At least 50 neurons were scored for each condition. *P⩽0.01, **P⩽0.001. Calibration bar=20 μm.

To study the possible involvement of selected SNARE family proteins on the regulation of initial axonal outgrowth and the establishment of neuronal polarity, we silenced the expression of such proteins using targeted shRNAs inserted into dicistronic plasmids also encoding enhanced green fluorescent protein (GFP). Transfection of cell cultures with VAMP4-targeted shRNA significantly and specifically decreased VAMP4 protein (Figure 2b). The transfected neurons expressed virtually no detectable VAMP4 and failed to form axons; only short, minor neurites were present (Figure 2a, second row from top; arrow). Note also the lack of enrichment of tau-1 in any neurite of the transfected neurons (Figure 2a-second row from top-arrow). In contrast, neurons transfected with a scrambled RNA sequence inserted in the same plasmid exhibited normal levels of VAMP4 and generated a long axon-like process enriched in tau-1 protein (Figure 2a, top). The same result was observed in non-transfected neurons (second row from top, arrowhead). Co-transfection of neurons with VAMP4-targeted shRNA and a myc-tagged wild-type form of VAMP4 (human) rescued the phenotype and induced the outgrowth of an axon-like process enriched in tau-1 protein (Figure 2a, bottom). To analyze this observation quantitatively we scored the differentiation stages of neurons transfected with VAMP4-targeted shRNA compared to neurons in the same cultures not containing shRNA, after 24 h *in vitro*. We found that over 70% of the transfected neurons remained at stages 1 or 2 of differentiation, and <30% had formed a discernible axon. In contrast, ~70% of the control neurons showed an identifiable, tau-1-containing axon (Figure 2c). We performed similar experiments with shRNA targeting Syntaxin6 and SNAP23. Western blots (WBs) showed that transfections with the dicistronic plasmids containing either shRNA targeted to Syntaxin6 or SNAP23 significantly reduced Syntaxin6 expression (Figure 2e) or SNAP23 expression (Figure 2h) respectively in cell cultures. Syntaxin6 was near detection limit (Figure 2d second row from top) and SNAP23 was not detectable (Figure 2g second row from top, arrows) in the transfected neurons. Most of such neurons failed to form an axon and remained in stage 2 of differentiation (Figure 2d for shRNA to Syntaxin6 and Figure 2g for shRNA to SNAP23, respectively). These neurons also failed to polarize tau-1 to any process, whereas growing axons from ssRNA-transfected or non-transfected neurons in the same culture were clearly tau-1-positive (Figure 2d-top and 2G-top, respectively). As in the case of VAMP4 most neurons transfected with Syntaxin6-targeted shRNA (Figure 2f) or SNAP23-targeted shRNA (Figure 2i) remained in stages 1 and 2 of differentiation. Co-transfection with Syntaxin6-targeted shRNA plus a wild-type form of Syntaxin6 (human-Figure 2d-bottom) or with SNAP23-targeted shRNA plus a wild-type form of SNAP23 (mouse-Figure 2g-bottom) rescued the phenotype and generated neurons bearing axon-like processes enriched in tau-1 protein. Taken together, this group of results suggest that VAMP4, Syntaxin6 and SNAP23 are necessary for initial axonal outgrowth and the establishment of neuronal polarity. Contrasting results were found when we silenced VAMP2 or VAMP7. WBs showed that transfection with the dicistronic plasmid containing VAMP2-targeted shRNA plus GFP significantly reduced VAMP2 expression in cell cultures (Figure 3c). VAMP2 was not detectable by immunofluorescence (IF) in transfected neurons (Figure 3a bottom, arrow). Suppression of VAMP2 did not affect neuronal polarization (Figure 3c). Since VAMP2 function could be compensated by VAMP1 or VAMP3, we measured neuronal polarization in cultures treated with tetanus toxin (an inhibitor of exocytosis triggered by VAMP1, VAMP2 and VAMP3) [30, 31]. Our results showed that incubation with 50 nM tetanus toxin did not preclude neuronal polarization (Supplementary Figure S2). Also, transfection of cell cultures with VAMP7-targeted shRNA significantly and specifically decreased VAMP7 protein in the cultures (Figure 3f). The transfected neurons expressed virtually no detectable levels of VAMP7 (Figure 3d bottom-arrow) and generated a relatively long axon-like process enriched in tau-1 protein and exhibited a similar morphology as control, ssRNA-transfected neurons (Figure 3d top). To analyze this observation quantitatively we scored the differentiation stages of neurons transfected with VAMP7-targeted shRNA compared to neurons in the same cultures not containing shRNA, after 24 h *in vitro*. We found that there are not significant differences in the number of neurons in stage 3 between the neurons transfected with VAMP7-targeted shRNA and those transfected with ssRNA (Figure 3e). Since VAMP7 has been proposed to participate in neurite elongation in cortical neurons cultured on laminin [28], we also analyzed polarization and axonal length in hippocampal neurons cultured on laminin for 24 or 48 h. Our results showed that suppression of VAMP7 by shRNA did not inhibit neuron polarization (Figure 3g) but significantly inhibited axonal elongation (Figure 3h). We performed similar experiments with shRNA targeting VAMP2. WBs showed that transfection with the dicistronic plasmid containing VAMP2-targeted shRNA plus GFP significantly reduced VAMP2 expression in cell cultures (Figure 3c). VAMP2 was not detectable by IF in transfected neurons (Figure 3a bottom, arrow). As for VAMP7, suppression of VAMP2 did not affect neuronal polarization (Figure 3c).

**Figure 4:**
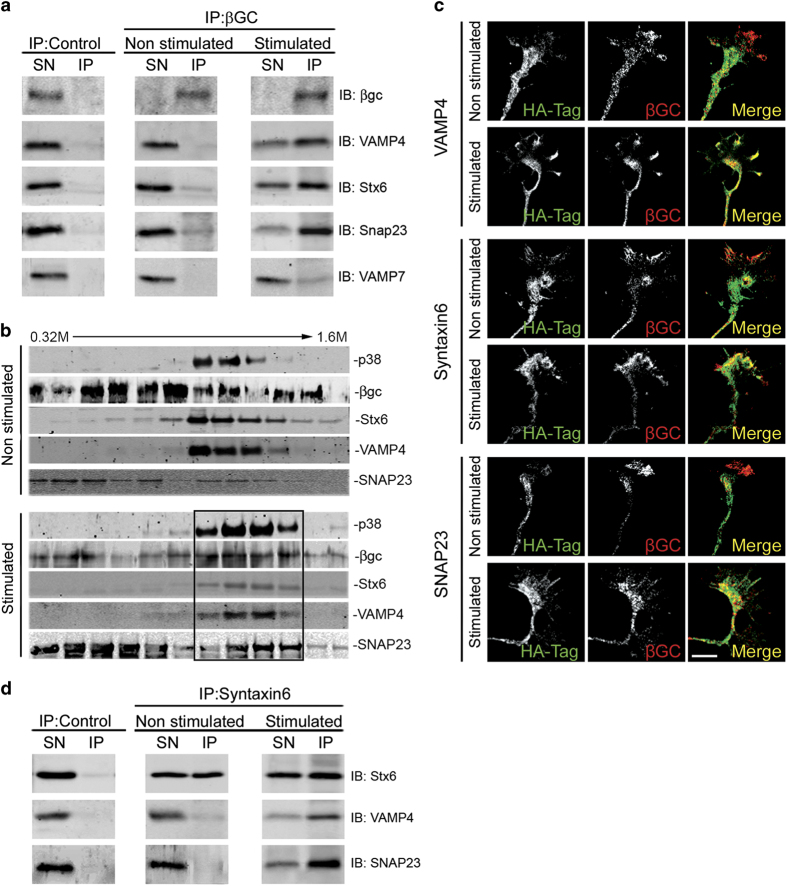
(**a**) Western blots of lysed GCPs (containing resealed PPVs element belonging to the plasma membrane) immunoprecipitated in the absence of any relevant antibody (left) or with the anti-IGF-1 receptor antibody βgc (middle and right). Before lysis the GCPs were kept in control medium (left and middle) or challenged with 20 nM IGF-1 for 5 min (right). The blots were probed with the following antibodies: βgc, anti-VAMP4, anti-Syntaxin6, anti SNAP23 and anti-VAMP7 respectively (from top to bottom) SN=Supernatant, IP: Immunoprecipitate. (**b**) Lysed GCPs in control non-stimulated conditions or stimulated for 5 min with 20 nM IGF-1 separated across a continuous sucrose gradient extending from 0.3 to 1.6 M sucrose by isopincnic centrifugation (242 *g* for 2 h). Note the precise co-localization of the IGF-1 receptor (βgc), VAMP4, Syntaxin6, SNAP23 and the vesicles marker p38 in the stimulated samples (bottom-box). (**c**) Immunofluorescence micrographs showing the distribution of βgc at the growth cone of pyramidal neurons in culture HA was used as a transfection control. Neurons were transfected with HA-tagged wt-VAMP 4 (first and second row), HA-tagged wt-Syntaxin6 (third and fourth row) or HA-tagged SNAP23 (fifth and sixth row) and kept in control medium (first, third and fifth row) or challenged with 20 nM IGF-1 for 5 min. Note that stimulation with IGF-1 promotes colozalization of the three SNARE proteins assayed with the IGF-1 receptor. (**d**) A) Western blots of lysed GCPs (containing resealed PPVs) immunoprecipitated in the absence of any relevant antibody (left) or with anti-Syntaxinn6 antibody (middle and right). Before lysis the GCPs were kept in control medium (left and middle) or challenged with 20 nM IGF-1 for 5 min (right). The blots were probed with the following antibodies: anti-VAMP4, anti-Syntaxin6 and anti SNAP23 respectively (from top to bottom). IP, immunoprecipitate; SN, supernatant. Scale bar, 2 μm.

An early event of axonal specification during neuronal differentiation is the enrichment of activatable IGF-1 receptor in one minor neurite at stage 2 of differentiation [1]. In order to be activated, the IGF-1 receptor needs to be inserted into the neuronal plasmalemma, so that the ligand-binding site is exposed to the extracellular space. Moreover, exocytotic insertion of IGF-1 receptor into the neuronal plasmalemma is activated by its cognate ligand, IGF-1 [14]. Therefore, we studied the possible involvement of the SNARE proteins VAMP4, Syntaxin6 and SNAP23 on the polarized exocytosis of vesicles containing the IGF-1 receptor. We first investigated the association of selected SNARE proteins with vesicles containing the IGF-1 receptor in growth cone particles (GCPs) isolated from rat fetal brain (GCPs; [32]). By immunoprecipitation of vesicles from control lysed GCPs with the anti-IGF 1 receptor antibody βgc (see Methods) we did not find a noticeable association of VAMP4, Syntaxin6, SNAP23 or VAMP7 with the immunoprecipitated vesicles (IP-Figure 4a-middle). Upon a challenge with 20 nM IGF-1 we found a strong association of VAMP4, Syntaxin6 and SNAP23 with the IP (Figure 4a right, second, third and fourth row from top). In contrast, little or no association with the IP was found for VAMP7 (Figure 4a right, bottom), a SNARE not necessary for neuronal polarization (see above). Lysed GCPs contain vesicles plus resealed elements formed from plasma membrane. To characterize the membrane structures containing the IGF-1 receptor plus VAMP4, Syntaxin6 and SNAP23 lysed GCPs (in non-stimulated conditions or stimulated with 20 nM IGF-1 for 5 min) were fractionated by isopicnic centrifugation in lineal sucrose density gradients and analyzed by WBs with antibodies against the IGF-1 receptor (βgc), VAMP4, Syntaxin6, SNAP23 and the vesicular marker p38. Results are shown in Figure 4b and show that in non-stimulated conditions, there was no noticeable co-migration of the analyzed proteins in the gradients. However, stimulation with IGF-1 caused a shift of the IGF-1 receptor to heavier fractions and an almost exact co-localization of IGF-1 receptor and the analyzed SNARE proteins plus the vesicle marker p38 was evident (Figure 4b-bottom-box). These results indicate that the challenge IGF-1 triggered the association of the IGF-1 receptor and the SNAREs in the same population of vesicles. Consistent with these results, a noticeable co-localization of VAMP4 (Figure 4c, second from top), Syntaxin6 (Figure 4c, fourth from top) and SNAP23 (Figure 4c, bottom) with the IGF-1 receptor recognized by βgc antibody was found at the growth cone of neurons stimulated with 20 nM IGF-1, compared with the respective, non-stimulated control cells (Figure 4c, top; Figure 4c, third from top; and Figure 4c fifth from top, respectively). Moreover, our results showed that stimulation with IGF-1 promoted the association of VAMP4 and SNAP23 with vesicles containing Syntaxin6 (Figure 4d).

**Figure 5:**
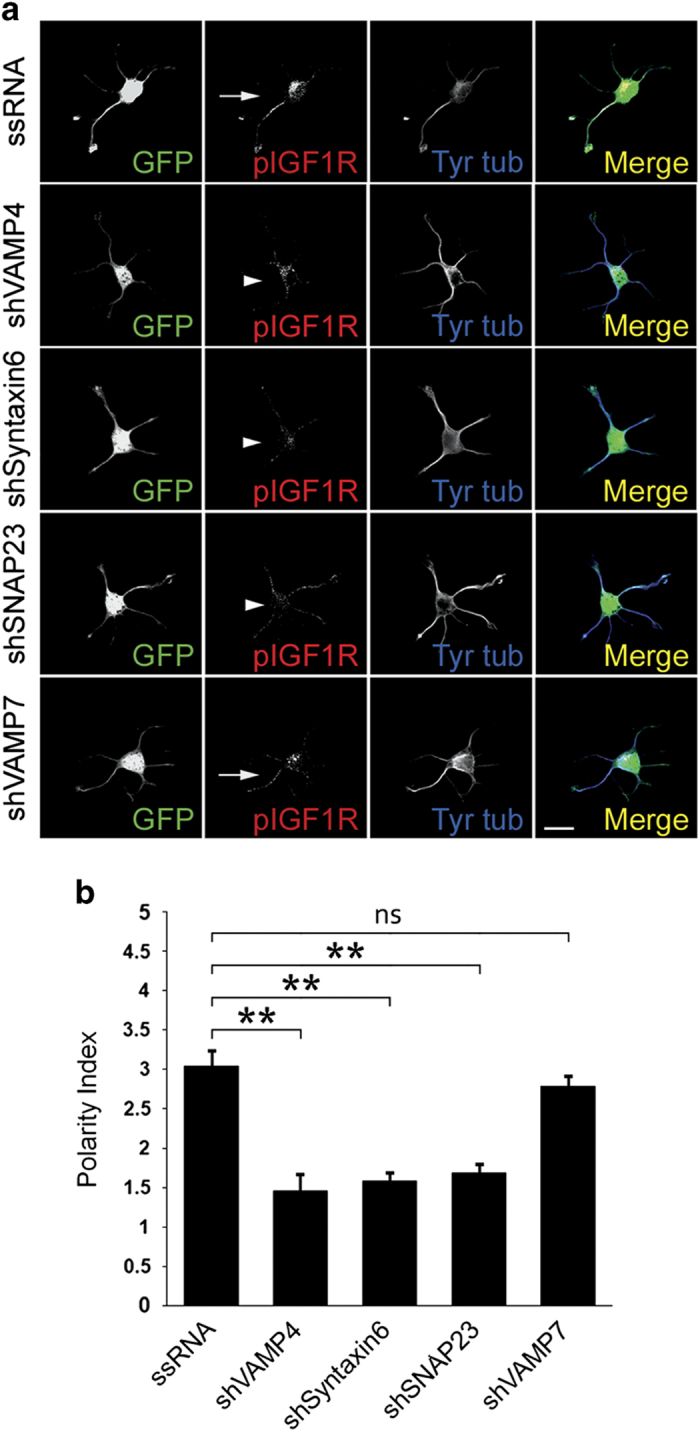
(**a**) Double immunofluorescence micrographs of hippocampal neurons (14 h in culture) that show the distributions of tyrosinated α-tubulin (blue), phosphorylated IGF-1 receptor (red), and the transfection marker GFP. Neurons were transfected with either a scrambled RNA sequence (ssRNA-top), VAMP4-targeted shRNA (second row), Syntaxin6-targeted shRNA (third row), SNAP23-targeted shRNA (fourth row) or VAMP7-targeted shRNA (bottom). Neurons were deprived of growth factors for 4 h and stimulated with 20 nM IGF-1 before fixation. Note the polarization of active (membrane-inserted) IGF-1 receptor to one of the minor neurites of the cell transfected with ssRNA (arrow-top) or transfected with VAMP7-targeteed shRNA (bottom). In contrast, neurons transfected with VAMP4, Syntaxin6 or SNAP23 (arrowheads-second, third and fourth rows fail to polarize the active IGF-1 receptor. (**b**) A ‘polarization index’ of active IGF-1 receptor (IGF-1r P.I.) was calculated as the fluorescence intensity (A.U.) of the brightest minor neurite/average fluorescence intensity (A.U.) of the other minor neurites of the same cell. Neurons were processed as in A). The polarization index is significantly higher in the neurons transfected with scrambled sequence RNA (*P=<0.01) compared to those transfected with VAMP4-, Syntaxin6- or SNAP-23-targeted shRNA. No significant differences in the polarization index were found in the neurons transfected with ssRNA and VAMP7-targeted shRNA. Scale bar, 20 μm.

We next studied the consequences of loss of function of VAMP4, Syntaxin6 or SNAP23 on the polarization of activated, that is, phosphorylated IGF-1 receptor to one neurite in stage 2 neurons (monospecificity of this antibody under our experimental conditions has been demonstrated previously; [1]). In stage 2 neurons transfected with a scrambled RNA sequence (14 h *in vitro*), deprived of growth factor for 2 h, and stimulated for 2 min with 20 nM IGF-1, we observed the expected polarized distribution of the activated IGF-1 receptor (Figure 5a top). Similar results were observed in cells transfected with VAMP7-targeted shRNA (Figure 5a bottom). In contrast, neurons transfected with VAMP4 (Figure 5a-second from top) or Syntaxin6 (Figure 5a-third from the top) or SNAP23 (Figure 5a-fourth row from the top) targeted shRNA exhibited labeling of the activated IGF-1 receptor that was less intense and not confined to any particular minor process. To quantify these differences we calculated an ‘active IGF-1 receptor polarization index’ (see legend to Figure 5b). As shown in Figure 5b this index was significantly higher (*P*=0.001) in the neurons transfected with the scrambled RNA sequence than in the VAMP4, Syntaxin6 or SNAP23-suppressed neurons.

**Figure 6:**
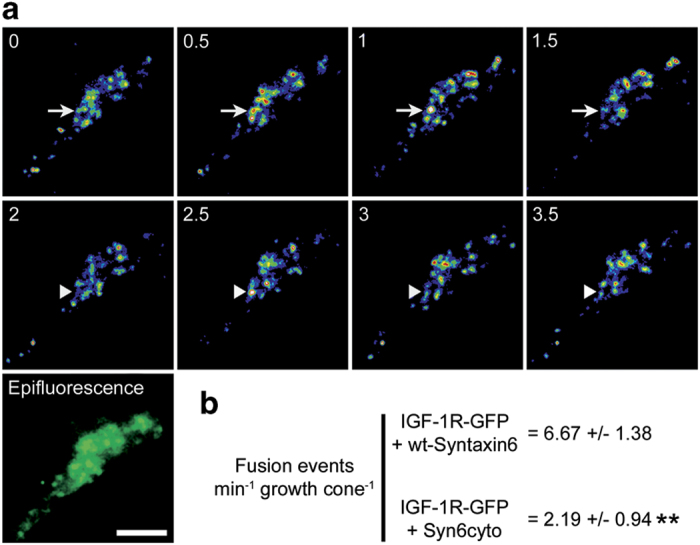
(**a**) Time-lapse TIRF images showing two examples of fusion events in the axonal growth cone of a hippocampal pyramidal neuron transfected with wild-type Syntaxin6 plus IGF-1 R-GFP. Vesicles that undergo fusion are marked with arrows (top) or arrowheads (middle). An epifluorescence image of analyzed growth cone was also included (bottom). Images were recorded at 0.2-second intervals, for 2 min. (**b**) Numbers represent the amount of fusion events per min per growth cone at the growth cone of neurons transfected with wild-type Syntaxin6 or negative dominant Syntaxin6. **P⩽0.001. Calibration bar= 2 μm.

In order to establish a direct relationship between Syntaxin6 activity and the exocytosis of PPVs containing the IGF-1 receptor at the growth cone of differentiating hippocampal neurons, we used time-lapse total internal reflection fluorescence (TIRF) microscopy [14, 33] to evaluate whether or not transfection with Syn6cyto, a dominant-negative form of Syntaxin6 [34] reduced the number of fusion events of IGF-1 receptor-GFP-containing vesicles with the growth cone plasmalemma. Figure 6 shows three examples of such fusion events at the growth cone of a hippocampal pyramidal neuron transfected with wt-Syntaxin6 (see also Supplementary movies S1 and S2 showing TIRFM time lapses of growth cones from neurons transfected with wt-Syntaxin6 or Syn6cyto, respectively) plus IGF-1 receptor-GFP (PS-GFP(LT)IGF-1R, see Methods). The estimated frequency of fusion events at the growth cone of neurons transfected with wt-Syntaxin6 plus IGF-1 receptor-GFP was 6.67 min-1 growth cone-1 (*n*=16) compared with 2.19 min-1 growth cone-1 (*n*=12) for neurons transfected with Syn6cyto plus IGF-1 receptor-GFP. These results demonstrate that Syntaxin6 is necessary for the exocitosis of IGF-1 receptor-containing vesicles to the plasmatic membrane of the growth cone.

## Discussion

During differentiation, neurons must enlarge their surface rapidly to support axonal outgrowth. This necessitates recruitment of newly synthesized membrane to the cell surface, by exocytotic insertion of PPVs at the growth cone [10]. Earlier studies from our laboratory demonstrated that IGF-1 stimulates PPV exocytosis at the axonal growth cone [8]. This occurs via activation of a receptor isoform that contains the immunochemically distinct βgc subunit [35, 36] and requires the activation of the phosphatidyl inositol 3-kinase-Akt signaling pathway [9]. This cascade, essential for the regulation of membrane expansion in developing neurons, also involves the activation of the small GTPase TC10 and the exocist complex [14]. Besides the exocyst complex, PPVs exocytosis require the activity of proteins belonging to the SNARE family. The components of this family involved in the regulation of exocytosis necessary for initial axonal outgrowth and the establishment of neuronal polarity have not yet been identified. This is the main theme of the present report.

Published data indicate the participation of several SNARE proteins in neurite outgrowth (see Introduction). The goal of the experiments in the present report was to identify SNARE proteins specifically involved in the early regulation of initial axonal outgrowth and, therefore, necessary for the establishment of neuronal polarity. We first investigated the temporal expression in developing neurons of seven SNARE proteins VAMP2, VAMP4, VAMP7, Syntaxin1, Syntaxin6 and SNAP23. Our results show that five of these SNAREs (VAMP2, 4 and 7, Syntaxin6 and SNAP23) are expressed in neurons in stage 2 before polarization. Silencing experiments using SNAREs-targeted shRNA indicated that three of these SNAREs are necessary for neuronal polarization; VAMP4, Syntaxin6 and SNAP23 (together with TC10 and the exocyst complex, [14]). Previously published results indicate that both Syntaxin6 and SNAP23 are also involved in the exocytosis of the Glu-T4 transporter in adypocites, a process that require stimulation with insulin and the participation of the exocyst complex [37–40]. In contrast, silencing experiments using a shRNA targeted to VAMP2 or treatment of hippocampal pyramidal neurons with tetanus neurotoxin did not preclude neuron polarization. These results could apparently contradict published information indicating that VAMP2 participates in neurite elongation in cortical neurons containing Apo4-Mito or FP4-Mito growing on laminin [28]. However, the studied models are different and there is published information from other authors indicating that VAMP2 is not required for axonal elongation in cultured hippocampal neurons [25]. Regarding VAMP7 our results indicate that the suppresssion of this SNARE does not affect significantly neuronal polarization in hippocampal neurons growing on polilysin or laminin but inhibits axonal outgrowth in cells growing on laminin, these results closely resemble those found in cultured hippocampal neurons from VAMP7 knockout mice [25, 41]. In summary, our results and the analysis of previous published work indicate that VAMP4, Syntaxin6 and SNAP23 are involved in the regulation of initial axonal outgrowth necessary for hippocampal neuron polarization. VAMP2 and VAMP7 can be involved in neurite elongation in different systems but do not seem to participate in the regulation of initial axonal outgrowth and the establishment of neuronal polarity in hippocampal neurons.

We have shown previously that IGF-1 and its receptor, which regulate exocytosis of PPVs at the axonal growth cone, are essential for the establishment of neuronal polarity [1]. Activation of the IGF-1 receptor requires its insertion into the plasmalemma. By probing for the appearance of activated IGF-1 receptor in undifferentiated neurites we demonstrated that the exocyst is necessary for IGF-1 receptor externalization in non-polarized neurons [14]. It follows, therefore, that insertion of IGF-1 receptor in an undifferentiated neurite (stage 2 hippocampal pyramidal neurons) is necessary for polarization. We performed several experiments to study a possible relationship between VAMP4, Syntaxin6 and SNAP23 activity and the polarized insertion of the IGF-1 receptor. Our results indicated that upon stimulation with IGF-1 VAMP4, Syntaxin6 and SNAP23 interact with vesicles containing the IGF-1 receptor. Moreover, IGF1 promotes the association of VAMP4 and SNAP23 to vesicles containing Syntaxin6. Interestingly, recently published results show the expression of VAMP4 at the axonal growth cone of developing neurons and suggest co-localization of VAMP4 and the IGF-1 receptor in the same PPVs population [20]. Loss of function experiments show that the expression of VAMP4, Syntaxin6 or SNAP23 is required for the polarized insertion of the IGF-1 receptor in neurons of stage 2 (not yet polarized). We also propose that stimulation with IGF-1 triggers the assembly of a population of vesicles containing these three SNARES plus the IGF-1 receptor. It is known that Syntaxin 6 contains a Q motif homologous to Qc (the Q motif of SNAPS) and not the Qa motif typical of syntaxins [42]) and formation of SNARE complexes including Syntaxin 6 and SNAPS has not yet been demonstrated. It follows that, at this point, we cannot assert if the three SNAREs involved in the establishment of neuronal polarity acts through the formation of one or different SNARES complexes. Finally, time-lapse TIRF microcoscopy of hippocampal neurons growth cone demonstrate a direct involving of Syntaxin6 in the exocytotic insertion of IGF-1 receptor to the growth cone plasmalemma.

Taken together, our results indicate that VAMP4, Syntaxin6 and SNAP23 are required for the polarized insertion of the IGF-1 receptor (and probably other growth factor receptors such as TrkB [43]) and, therefore, are essential for the regulation of initial axonal outgrowth and the establishment of neuronal polarity. More investigation will be needed to study if other proteins belonging to the SNARE family can also participate in these phenomena and the precise mechanism by which these three SNAREs regulate the establishment of neuronal polarity.

## Materials and Methods

### Short hairpin RNA plasmids

The shRNA sequences used as targets were as follow: VAMP2, 5′-gcacctcctccaaatctta-3′; VAMP4, 5′-gaatattaccaaggtaatt-3′; VAMP7, 5′-ggacaggattgtgtatctt-3′; Syntaxin6, 5′-gaacaatctccgcagcata-3′; SNAP23, 5′-ggaatcaagactatcacta-3′. For controls, a sequence of scrambled shRNA was used: 5′-gtgtatgattaggtaacgg-3′. The resulting plasmids were referred as shVAMP2, shVAMP4, shVAMP7, shSyntaxin6, shSNAP23 and ssRNA (scrambled shRNA), respectively. All oligonucleotides were synthesized by SIGMA (Sigma-Aldrich, St. Louis,MO, USA) and subcloned into pSuperNeo+GFP vector (Oligoengine).

### Dna constructs

To generate HA-tagged VAMP4 full length construct the coding sequences of VAMP4 full length was cut in EcoRI/BamHI flanking sites and cloned into EcoRI/BglII pCMV-HA vector (Clontech Laboratories Inc., Mountain View, CA, USA). The GFP-tagged full length human VAMP4 (GFP-VAMP4 FL) construct was a generous gift from Dr Marc Coppolino [44]. HA-tagged human full length Syntaxin6 (Syntaxin6-HA) and Myc-tagged cytosolic domain of human Syntaxin6 (Myc-Syn6cyto) constructs were kindly provided by Dr Sima Lev [45] and Dr Nobuhiro Nakamura [34], respectively. Syntaxin6 constructs used for TIRF experiments were generated as follow: in Syntaxin6 mCherry the coding sequence of rat Syntaxin6 was PCR amplified with forward primer 5′-aagctcagatctgaagaggacttgtccatg-3′ and reverse primer 5′-aacttccagggccaggagagg-3′ using as template Myc-tagged rat full length Syntaxin6 (Myc-Syntaxin6 pCMV4), a construct kindly provided by Dr Peter Arvan [46]. The PCR product was cloned into the BglII/HindIII sites of pmCherry C1 vector (Clontech). For Syn6cyto RFP the coding sequence of Myc-Syn6cyto construct was cut in HindIII/EcoRI flanking sites and cloned into HindIII/EcoRI of a pERFP vector. To generate HA-tagged SNAP23 two steps of cloning were required. First, the coding sequence from GFP-tagged mouse full length SNAP23 plasmid was cut in HindIII/XbaII flanking sites and cloned into HindIII/XbaII of pBKSII+ vector (StrataGen, Kirkland, WA, USA). SNAP23-GFP plasmid was a generous gift from Dr Giulia Baldini [47]. After that, the pBKSII+SNAP23 construct was cut in SalI/NotI and cloned in pCMV-HA vector (Clontech).

For TIRF experiments a plasmid encoding IGF-1 receptor coupled with GFP was designed. In this construct, GFP molecule is joined to alpha chain of IGF-1R through a hinge (in order to avoid esteric effect in the ligand-binding site of receptor) and a thrombin site to remove the fluorescent reporter before performing exocytosis experiments. Construction involved the following steps: first, GFP casette from pEGFP C1 vector (Clontech) was PCR amplified using forward primer 5′-gcagagctggtttagtgaaccgtcagatccgctagcgctaccggtcgccaccatg-3′ and reverse primer 5′-cgtatagatctcgatcctcggggtactagaggtcctggtcccttgtacagctcgtccatgcc-3′, the later coding a Gly-Pro-Gly-Pro linker (L) (this linker minimize the conformational changes in tertiary structure and provide flexibility of the peptide chain, [48]) plus a thrombin site (T). The PCR product was cloned into the NheI/BglII sites of a pEGFP C1 vector used as backbone and GFP(LT) C1 plasmid was obtained. Second, two oligonucleotide complementary sequences encoding the signal peptide of IGF-1R, forward strand 5′-ctagccgccaccatgaagtctggctccggaggagggtccccgacctcgctgtgggggctcctgtttctctccgccgcgctctcgctctggccgacgagtggagaa-3′ and reverse strand 5′-ccggttctccactcgtcggccagagcgagagcgcggcggagagaaacaggagcccccacagcgaggtcggggaccctcctccggagccagacttcatggtggcgg-3′ were annealed and cloned into AgeI/NheI sites of pEGFP(LT) C1 plasmid, so PS-GFP(LT) C1 was generated. Third, coding sequence of human IGF-1 R β-chain and part of α-chain from CVN-IGF-1 R construct were cut in KpnI/BamHI and cloned into KpnI/BamHI sites of PS-GFP(LT) C1. CVN-IGF-1 R plasmid was kindly provided by Dr Renato Baserga [49]. Finally, the remaining region of IGF-1 R α-chain from CVN- IGF-1 R construct was PCR amplified using forward primer 5′-gacgagtcgacaaatctgcgggccaggcatcg-3′ and reverse primer 5′- cggtgaagctgatgagatccctgtagtc-3′. The PCR product was cut in SalI/KpnI flanking sites and cloned into the XhoI/KpnI sites of PS-GFP(LT)ΔIGF-1 R C1 plasmid. So, PS-GFP(LT)IGF-1 R was obtained. All the constructs were sequenced.

### Primary antibodies

The following primary antibodies were used: rabbit polyclonal antibody to VAMP2 (ABCAM), diluted 1:500 for IF and 1:1000 for WB; mouse monoclonal antibody to VAMP2 (Synaptic System), diluted 1:1 000 (IF) and 1:3 000 (WB); rabbit polyclonal antibody to VAMP4 (Synaptic System, Goettingen, Germany), diluted 1:100 (IF) and 1:250 (WB); affinity-purified mouse monoclonal antibody to VAMP7/SYBL1 (clone 158.2, ABCAM, Cambridge, MA, USA), diluted 1:100 (IF) and 1:250 (WB); affinity-purified mouse monoclonal antibody to Syntaxin1 (clone 78.2, Synaptic Systems), diluted 1:150 (IF) and 1:400 (WB); rabbit polyclonal antibody to Syntaxin6 (Synaptic System), diluted 1:150 (IF), 1:600 (WB) and 1:250 (IP); rabbit polyclonal antibody to SNAP23 (Synaptic System), diluted 1:100 (IF) and 1:200 (WB); affinity-purified mouse monoclonal antibody to SNAP25 (clone SP12, StressGen Biotechnologies, Victoria, British Columbia, Canada), diluted 1:100 (IF) and 1:200 (WB); rabbit polyclonal antibody to βgc [35], diluted 1:100 (IF), 1:200 (WB) and 1:150 (IP); rabbit polyclonal antibody to phosphorylated (Tyr 980) IGF-1r (Cell Signaling), diluted 1:50 (IF); rabbit polyclonal antibody to βIII-tubulin (Sigma-Aldrich), diluted 1:4000 (IF); mouse monoclonal antibody to the axonal marker Tau-1 (clone PC1C6, EMD Millipore, Darmstad, Germany), diluted 1:600 (IF); rat monoclonal antibody to tyrosinated tubulin (clone Tub-IA2, Sigma-Aldrich), diluted 1:2 000 (IF); mouse monoclonal antibody to α-tubulin (clone DM A1, Thermo Scientific, Waltham, MA, USA), diluted 1:2000 (WB); rat monoclonal antibody to HA (clone 3F10, Roche, Basel, Switzerland), diluted 1:400 (IF); and rabbit monoclonal antibody to p38 (Abcam) diluted 1:1 000.

### Culture and transfection

Dissociated hippocampal pyramidal neurons were prepared from fetal rat brain and cultured as described previously [50]. In brief, cells were plated onto polylysine (or laminin (10 μg ml^−1^ in the experiments shown in Figures 3g and h)-coated glass coverslips and maintained in Dulbecco’s modified Eagle’s medium (DMEM) plus 10% horse serum for 1 h. The coverslips with the attached cells were transferred subsequently to 35 mm Petri dishes containing serum-free medium plus the N_2_ mixture. Cultures were maintained in a humidified 37 °C incubator with 5% CO_2_. Shortly after plating, hippocampal neurons first extend lamellipodia (stage 1) and afterward several minor neurites that are initially indistinguishable (stage 2). Then, at stage 3, one of these initially equivalent neurites grows more rapidly than the others and becomes the axon, whereas the other neurites subsequently develop into dendrites (stage 4). Neurons are considered to be at stage 3 when the length of the axon exceeds that of the average minor neurite by at least 20 μm [29]. Transient transfection of cultured neurons was performed as described previously [50], and the constructs used at a concentration of 2.5 μg μl^−1^.

For those experiments involving expression of shRNA sequences in early stages of neuronal development, a protocol of transfection of neurons in suspension, before plating, was used. It was similar to *in vitro* procedure described in [51], with modifications. Briefly, DNA:Lipofectamine 2000 complex diluted in OPTIMEM (80 μl) was made in a 1.5 ml Eppendorf tube. Usually, 500 ng of DNA and 1 μl of Lipofectamine 2000 were mixed in each reaction. This mixture was incubated at room temperature for 30 min and after that 15 μl of a neuron suspension (6×10^4^ cells) diluted in OPTIMEM was added. Cells-DNA-Lipofectamine 2000 mixture was immediately plated over polylysine-coated glass coverslips and cultures were placed to 37 °C in a humidified 5% CO_2_ incubator. After 1 h, transfecting complex was removed carefully from each coverslip (at this time most of neurons were already attached) and serum-free medium plus the N_2_ mixture was added to cultures. Cultures were maintained in a humidified 37 °C incubator with 5% CO_2_ for 24–48 h before fixation. So, detectable levels of silencing were achieved within 24 h of transfection with shRNA sequences.

### Immunofluorescence microscopy

Cells were fixed for 20 min at room temperature with 4% (w/v) paraformaldehyde in phosphate-buffered saline (PBS) containing 4% (w/v) sucrose. Cultures were washed with PBS, permeabilized with 0.2% (v/v) Triton X-100 in PBS for 6 min, again washed in PBS and blocked for 1 h at room temperature. After labeling with a first primary antibody (1–3 h at room temperature or overnight at 4 ºC) and washing with PBS, cultures were incubated with fluorescent secondary antibody conjugated to Alexa Fluor 488, 546 or 633 (1 h at room temperature) and washed with PBS. The cells were visualized using a spectral confocal microscope (Olympus FV1000, Tokyo, Japan). Images were captured and digitized using Olympus Fluoview Viewer software. For some experiments (see Figure 6), cells were observed with an Olympus Spining Disk (DSU) microscope equipped for TIRF. Images were captured using a charged-coupled camera (Andor Ixon3, Andor Oxford Instruments, Oxfordshire, UK). Images were digitized using Olympus Fluoview Viewer software. In some cases, the images were analyzed using ImageJ (National Institutes of Health, Rockville, MD, USA) software. All images were processed using Adobe PhotoShop (Adobe Systems, San Jose, CA, USA).

### VAMP4, Syntaxin6 and SNAP23 rescue experiments

To perform these experiments, we titrated levels of VAMP4, Syntaxin6 or SNAP23 plasmid to determine the lowest concentration necessary to overcome the shRNA effects. Under these conditions, cells transfected with VAMP4, Syntaxin6 or SNAP23 shRNA, together with VAMP4, Syntaxin6 or SNAP23 plasmid, respectively, exhibited protein levels that were similar to those of control non-transfected neurons.

### Gel electrophoresis and western blot

Proteins were analyzed by SDS–polyacrylamide gelelectrophoresis. The concentration of acrylamide of the resolving gel varied from 7.5 to 15%. The resolved proteins were transferred to nitrocellulose membranes (Amersham Hybond-ECL, GE Healthcare, Buckinghamshire, UK) in Tris-glycine buffer containing 20% methanol. The membranes were first dried, washed with Tris-buffered saline (10 mM Tris, pH 7.5, 150 mM NaCl) and then blocked, or directly blocked for 1 h in Tris-buffered saline containing 5% bovine serum albumin. The blots were incubated with the primary antibodies in PBS containing 0.05% Tween 20, for 12 h at 4 ºC. After washing with Tris-buffered saline containing 0.05% Tween 20, the membranes were incubated with Odyssey IRdye CW 800 secondary antibodies (LI-COR Biosciences) for 1 h at room temperature. After washing, the blots were imaged using an Odyssey Infrared Imaging System (LI-COR Biosciences, Lincoln, NE, USA).

### Isolation of growth cones

Axonal growth cones were isolated from developing brain as described previously [32, 52]. In brief, brains of 18-days gestation fetal rats were homogenized (H). A low-speed supernatant was prepared, loaded onto a discontinuous sucrose density gradient with steps of 0.83, 1 and 2.66 M sucrose, and spun to equilibrium at 242000 *g* max. The fraction at the load/0.83 M interface (designated ‘A’) contained the isolated growth cones or GCPs.

### Immunoprecipitation assays

Intact GCPs were incubated for 15 min at 4 °C in the presence or absence of 20 nM IGF-1. Afterwards, 3 μl saponin 2%; 30 μl of intracellular buffer 10× (Hepes 20 mM, ClNa 5 mM, ClK 50 mM, Cl_2_Mg 3 mM); 3 μl ATP and 64 μl H_2_O were added and the mixture incubated for 5 min at 37 ºC. Both stimulated and control GCPs were lysed with ice cold lysis buffer (EDTA 0,5 mM; Tris-HCl pH:7,5 6 mM; Tritón X100 1% v/v; Nacl 150 mM; PMSF 1 mM; aprotinin and H_2_O). This relatively mild lysis buffer allows release of PPVs contained into GCPs. For immunoprecipitation, solution containing both PPVs and resealed GCPs was incubated with the indicated antibodies overnight at 4 °C before adding proteinA/G plus-coated beads (Protein A-Sepharose 4B Fast Flow—SIGMA). In order to avoid unspecific binding, a control condition of incubation without beads was added. After incubation immunoprecipitation mixtures were spun to 4 000 r.p.m during 10 min, supernatant (SN) and immunoprecipitated (IP) were separated and samples processed to further analysis by SDS–polyacrylamide gelelectrophoresis/WB.

### Immunofluorescence of active IGF-1r

Cells were cultured as previously described and transfected with VAMP4, VAMP7, Syntaxin6 or SNAP23 shRNA before plating (that is, in suspension, at time 0 of IVC). After 10 h in culture, cells were deprived of growth factors for 4 h and then stimulated for 5 min with 20 nM IGF-1, fixed and processed for IF using an antibody selective for the phosphorylated form of IGF-1r [1] or an antibody that recognize both the phosphorylated and non- phosphorylated forms of IGF-1r (anti-βgc, [35]).

### Time-lapse total internal reflection fluorescence microscopy

For time-lapse TIRF microscopy, cells were cultured in special Petri dishes [53]. Eighteen h after transfection with either wild-type Syntaxin6 (Syntaxin6-mCherry) or the dominant-negative form of Syntaxin6, Syn6cyto (Syn6cyto-RFP) plus PS-GFP(LT)-IGF1R, the dishes containing the attached cells (deprived of growth factors for 4 h) were placed in a Harvard microincubator model TC 202A located on top of the stage of an Olympus Spining Disk (DSU) microscope equipped for differential interference contrast, epifluorescence and TIRF. We used TIRF to image single vesicular insertion events under low-intensity conditions that minimize phototoxicity [14, 54]. Cells were visualized using an Olympus UApo 150X 1.45 AN numerical aperture objective, equipped for TIRF illumination using an Olympus laser digital system (solid state). Neurons were imaged in Neurobasal medium supplemented with 20 nM IGF-1 and 30 mM HEPES buffer, pH 7.2, and maintained at 37 °C. Time-lapse sequences were acquired at a continuous rate of 2 frames s^−1^ during 2 min using an Andor Ixon3 model x-8677 camera (CCD 201-20-1-122). Images were digitized using Olympus Fluoview Viewer software. Pyramidal neurons were selected by morphological criteria (in wide-field images) before imaging in TIRF mode. Live-cell images shown represent raw data with simple background subtraction of the averaged blank field intensity. In order to identify those cells transfected with either wild-type Syntaxin6-mCherry or Syn6cyto-RFP plus PS-GFP(LT)-IGF1R, epifluorescence pictures of the neurons were taken before time-lapse imaging experiments. Only the growth cones from co-transfected cells were scored for quantification.

### Animals

All animal procedures were done using approved protocols by the Board of Animal Welfare, School of Chemical Sciences, National University of Córdoba.
