# Supplementary figures and images for: Selected SNARE proteins are essential for the polarized membrane insertion of igf-1 receptor and the regulation of initial axonal outgrowth in neurons
Source: Cell Discov. 2015 Sep 1;1:15023–. doi: 10.1038/celldisc.2015.23 (PMC4860833; doi:10.1038/celldisc.2015.23)

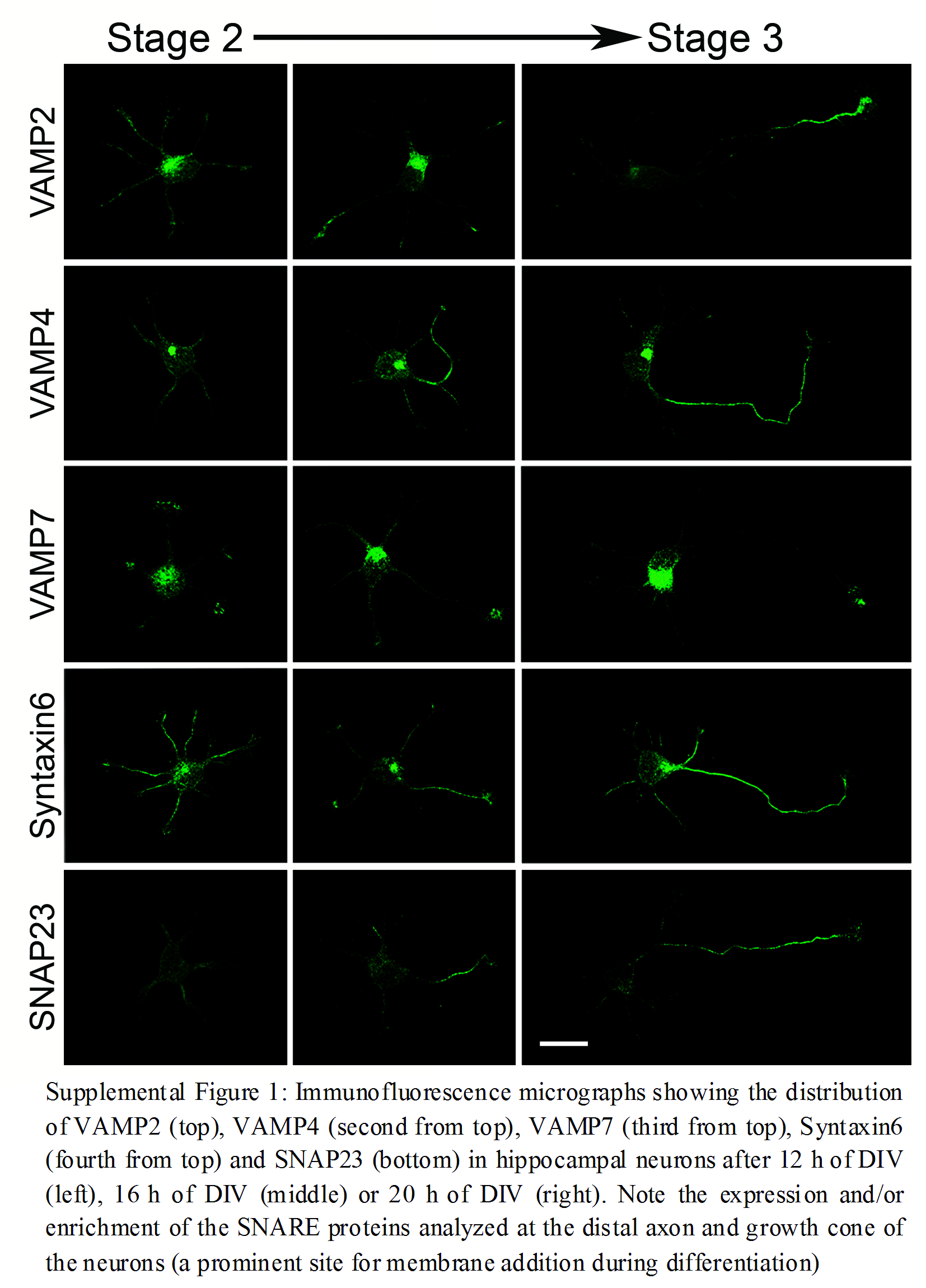

Supplement: Supplementary Figure 1 [file celldisc201523-s1.tiff]

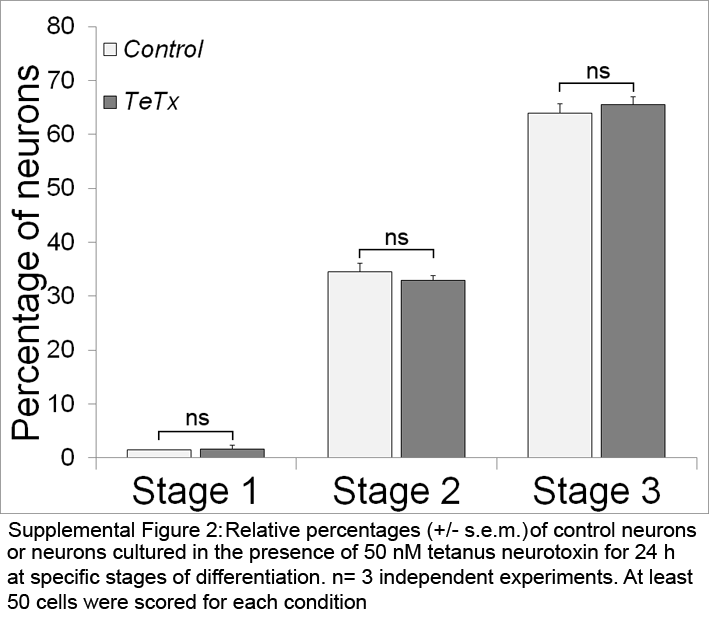

Supplement: Supplementary Figure 2 [file celldisc201523-s2.tiff]
